# Improving baseline and follow-up physical health monitoring when commencing oral antipsychotics

**DOI:** 10.1192/bjo.2021.279

**Published:** 2021-06-18

**Authors:** Nathan Mitchell, Aamer Sajjad, Anna Grocholewska-Mhamdi, Catherine McMain

**Affiliations:** 1 Hull York Medical School; 2 Navigo Heath and Social Care CiC

## Abstract

**Aims:**

NICE guidelines suggest baseline physical health monitoring be performed prior to commencing antipsychotics, in addition to follow-up monitoring for adverse effects for at least 12 months. ‘Shared Care Guidelines’ were adapted from NICE guidance for local use in North East Lincolnshire. Nevertheless, a local audit published in 2018 reported low compliance with baseline monitoring in community mental health teams (CMHTs) compared to inpatient teams. The parameter most infrequently performed overall was the Glasgow Antipsychotic Side Effect Scale (GASS) questionnaire.

This study aimed to assess whether compliance with baseline physical health monitoring had improved in line with the previous audit's recommendations. Additionally, it aimed to expand on previous findings by adding compliance data for follow-up physical health checks and produce further recommendations to optimise performance.

**Method:**

A retrospective re-audit was performed in NAViGO Health and Social Care to assess compliance with the guidelines for physical health monitoring when commencing antipsychotics in previously antipsychotic-naïve patients. Patient records were examined for which recommended physical health checks were performed at baseline, and at 1-, 3- and 6- months from commencing antipsychotics.

**Result:**

15 eligible patients were identified to have been commenced on antipsychotics, 8 patients under a CMHT and 7 under an inpatient team. The average overall compliance at baseline for checking 16 parameters was 50%. For the CMHT, compliance was 60%, compared to 38% for the inpatient team. Across both teams, baseline compliance was highest for renal function tests, liver function tests, and blood pressure and pulse (80%). For 1-, 3-, and 6- month checks, overall compliance for checking recommended parameters were 33%, 29% and 29% respectively. GASS monitoring compliance was 7% at baseline, 0% at 1- and 3-months, 7% at 6-months.

**Conclusion:**

The CMHT performed better than the inpatient team at baseline monitoring. This may reflect action on the previous audit's recommendations to increase provision of community ‘Wellbeing Health Improvement Service’ (WHISe) clinics. However, performance of the GASS questionnaire at baseline was consistent with the previous audit, with similar performance at follow-up extending these findings.

In response, the first recommendation is for Quality Improvement Activities to help improve compliance with the ‘Shared Care Guidelines’. This may include CQUINs and further provision of community clinics to improve compliance with both baseline and follow-up checks. Secondly, it is proposed that GASS questionnaires be sent to patients prior to appointments to be completed in advance to avoid further risk of GASS being incomplete.

